# Perceptions of the Use of Mobile Technologies for Smoking Cessation: Focus Group Study With Individuals of Low Socioeconomic Status Who Smoke

**DOI:** 10.2196/58221

**Published:** 2024-10-11

**Authors:** Michael Wakeman, Lydia Tesfaye, Tim Gregory, Erin Leahy, Brandon Kendrick, Sherine El-Toukhy

**Affiliations:** 1 Division of Intramural Research National Institute on Minority Health and Health Disparities National Institutes of Health Rockville, MD United States; 2 ICF Reston, VA United States

**Keywords:** smoking cessation, social determinants of health, mhealth, apps, qualitative research, young adults

## Abstract

**Background:**

The use of mobile technologies to deliver behavioral health interventions, including smoking cessation support, has grown. Users’ perceptions are important determinants of the adoption and use of new technologies. However, little is known about users’ perceptions of mobile technologies as smoking cessation aids, particularly among disadvantaged individuals who smoke.

**Objective:**

This study aimed to examine the acceptance of mobile technologies for smoking cessation among young adults with low socioeconomic status who smoke.

**Methods:**

In total, 38 current cigarette smokers, 18 to 29 years old, who wanted to quit and did not have a 4-year college degree nor were enrolled in a 4-year college, participated in 12 semistructured digital focus groups. The moderation guide was guided by the Unified Theory of Acceptance and Use of Technology. Discussions were audio recorded, transcribed verbatim, and coded for the Unified Theory of Acceptance and Use of Technology constructs (ie, effort expectancy, facilitating conditions, performance expectancy, and social influence), sentiment (ie, negative, neutral, and positive), and purpose of using mobile technologies (ie, lifestyle and health management and smoking cessation) following a deductive thematic analysis approach.

**Results:**

Participants had positive experiences using mobile technologies for lifestyle and health management, primarily for fitness and dietary purposes. Salient themes were facilitating conditions of use (44/80, 55%), with prior experiences and costs subthemes, followed by perceived usefulness of mobile technologies in helping users attain health goals (22/80, 27.50%), which were generally positive. Ease of use (11/80, 13.75%) and social influences (3/80, 3.75%) were minimally discussed. Conversely, participants had limited awareness of smoking cessation uses of mobile technologies, which was the primary barrier under facilitating conditions discussed (33/51, 64.70%). Participants expressed skepticism about the usefulness of mobile technologies in helping them quit smoking (14/51, 27.45%). Effort expectancy was not discussed, given participants’ limited prior use. Social influences on mobile technology use for smoking cessation were minimally discussed (4/51, 7.84%).

**Conclusions:**

The use of mobile technologies for smoking cessation was unknown to young adults with low socioeconomic status who smoke. To reduce cigarette smoking and associated health disparities, increasing awareness and use of evidence-based mobile-based smoking cessation interventions are needed. Smoking cessation interventions should incorporate features perceived as useful and easy to use to capitalize on positive user experiences and the acceptability of mobile technologies for lifestyle and health management.

## Introduction

Mobile apps, alone or in combination with sensors and wearables, allow for the delivery of personalized, around-the-clock smoking cessation support [1,2]. Mobile health interventions can complement or increase the reach of traditional smoking cessation support (eg, counseling and medication) as they overcome barriers to traditional smoking cessation aids that remain underused for various reasons such as stigma, insurance, and cost [3-8]. Accordingly, technology-based smoking cessation interventions are potentially beneficial to populations who are disproportionally affected by smoking such as individuals with low socioeconomic status (SES). Often measured by educational attainment, income, or combined indices, low SES is a determinant of smoking health disparities [9,10]. In the United States, 62.2% of adults 25 years or older had less than a college degree and 36.8% had a high school diploma or less [11]. Furthermore, 11.5% live in poverty according to 2022 data [12]. Individuals with low SES who smoke have higher-than-average smoking rates owing to individual and structural factors [13-15]. For example, 11.5% of American adults smoked cigarettes in 2021 [13]. However, the prevalence of cigarette smoking was highest among those who did not have a high school diploma or who earned a GED (20.1% and 30.7%, respectively) and among adults with low incomes (18.3%) [13]. Furthermore, low-income communities have been disproportionally exposed to and targeted by tobacco advertising [6,14,16]. Despite an interest in quitting among individuals with less than a high school education (68%) and those with low incomes (67.3%) [5], these populations are less likely to quit [5,6,13,14], receive cessation advice [5], and access cessation treatments [6,16,17].

With their high penetration, mobile technologies offer opportunities to increase the reach of smoking cessation interventions in general and to populations who are socioeconomically disadvantaged in particular [18]. At 85%, most American adults own smartphones, including those with low incomes (76%) and who are high school educated (75%) [18]. People are increasingly attached to and reliant on smartphones in their everyday lives, including for health and wellness [19,20]. For example, 49.24% of Americans have health apps on their phones, and 38.99% use their phones or tablets to track progress toward a health goal [21]. Researchers estimate there are over 200 smoking cessation apps available for smartphone users [22], although exact accounts of the number of smoking cessation apps, their downloads, and duration of use are largely unknown. Additionally, off-the-shelf sensors can track cigarette use (eg, smart lighters) and physiological parameters (eg, carbon monoxide) to aid smoking cessation [23,24]. Mobile smoking cessation apps and sensors are available to individuals who can self-initiate smoking cessation outside traditional health care settings or within health care systems through integration with clinical decision tools and e-referral programs [25,26].

The effectiveness of smoking cessation apps remains inconclusive [5]. A Cochrane meta-analysis showed no evidence of increased abstinence among individuals using smoking cessation apps compared with those receiving no or minimal cessation support (relative risk 1.00, 95% CI 0.66-1.52) [27]. Conversely, another meta-analysis found that smartphone interventions combined with pharmacotherapies were associated with higher smoking abstinence rates than pharmacotherapies alone (relative risk 1.79, 95% CI 1.38-2.33) [28]. Other studies have focused on cataloging the content and features of smoking cessation apps based on predefined criteria (eg, behavioral change techniques) and examining the associations between these features and user uptake, continued use, and quit success [1,29-32]. A limitation of the literature is the scarcity of studies on target users’ acceptance of these digital health tools and their willingness to use them as smoking cessation aids, which are precursors to app uptake and determinants of smoking cessation apps’ efficacy [33].

Theoretical frameworks such as the Technology Acceptance Model and the Unified Theory of Acceptance and Use of Technology underscore users’ perceptions of the adoption and use of new technologies [34,35]. Indeed, perceived usefulness and ease of use are antecedents for adopting digital health technologies [36]. Additionally, evidence shows the impact of barriers (eg, low awareness) and facilitators (eg, internal motivation), attitudes, and prior experiences and behavior on the uptake and continued use of digital technologies [37,38]. A recent systematic review on perceptions of smartphone apps for smoking cessation found that nearly all studies focused on specific apps and app functions (eg, tracking) rather than on acceptance of mobile technologies as smoking cessation aids in principle [39]. Additionally, only one study in this review focused on individuals with low SES who smoke [40]. Given the scarcity of studies on users’ perceptions of mobile technologies for smoking cessation, this study examined the acceptance of mobile technologies for smoking cessation among young adults with low SES who smoke.

## Methods

### Participants and Recruitment

A convenience sample of 38 young adults with low SES who smoke participated in 12 semistructured digital focus group discussions. Userworks, Inc (Silver Spring, MD) recruited participants between January and April 2020 through its research volunteer panels and commercial platforms (eg, Craigslist). Recruitment was not based on data saturation and no participants withdrew. Potential research volunteers received an email invitation to participate in the study and interested candidates answered eligibility questions over the phone. Those eligible were 18-29 years old and not 4-year college educated nor enrolled in a 4-year college as an indicator of low SES [9,10,41]. Those eligible were also current cigarette smokers who reported having smoked at least 100 cigarettes in their lifetime and reported current cigarette smoking every day or some days [42], willing to quit within 6 months, not currently using any smoking cessation aids and noncigarette combustible tobacco products (eg, cigarillos), owned a smartphone, and spoke English. Twelve participants participated in 2 focus group discussions.

### Procedures

Focus group discussions occurred over GoTo Meeting and lasted ≈1.5 hours. Discussions gauged participants’ smoking initiation and their perceptions of mobile technologies for lifestyle and health management generally and for smoking cessation particularly. Participants who lacked experience with mobile health technologies for smoking cessation could thus share their more likely experiences with mobile technologies for lifestyle and health management. The 32-item COREQ (consolidated criteria for reporting qualitative research) checklist appears in Table S1 in Multimedia Appendix 1 [43-48].

We conducted dry runs prior to the actual focus groups. TG, a user experience strategist and researcher (male), moderated discussions following a topic guide informed by technology acceptance models (Table S2 in Multimedia Appendix 1) [34]. EL, a strategic communications and marketing project director (female), acted as a back-up moderator and took notes. TG had training and certification in user research, including focus group discussions. The moderators had no prior relationship with the participants who were informed that the moderators were not affiliated with the research group that commissioned the study. TG, EL, and SEL were the only nonparticipants present during the calls. GoTo Meeting’s built-in auto transcription generated transcripts for each focus group, which were not returned to participants for comment. Three members of our staff verified all transcripts against audio files.

### Analysis

We used a deductive thematic analysis [49,50]. First, we reviewed the transcripts and a member of our team generated initial codes. Then, we developed a codebook and corresponding themes based on 4 Unified Theory of Acceptance and Use of Technology constructs: effort expectancy, facilitating conditions, performance expectancy, and social influence (Table 1) [35]. Additionally, we coded the transcripts for the sentiment (ie, negative, neutral, and positive) and purpose of using mobile technologies (ie, lifestyle and health management, smoking cessation). We introduced new codes when the data did not fit the a priori codes [49]. Codes were developed inductively for smoking behaviors and illustrative quotes were extracted (Note S1 and Table S3 in Multimedia Appendix 1). We applied a multicoding approach where multiple semantic domains could be applied to a quote but only one code could be selected from each domain [51], allowing us to capture conceptually independent meanings that could be reflected in one quote [49]. For example, only one of the technology acceptance codes could be used to label a quote. However, the same quote could be labeled with sentiment or technology use purpose codes.

MW and LT independently coded the transcripts and calculated inter-coder agreement in ATLAS.ti (version 8; ATLAS.ti Scientific Software Development GmbH). The intercoder agreement was acceptable. Krippendorf c-α binary was 0.85, showing high intercoder agreement in identifying relevant from irrelevant content. Krippendorf cu-α, which relates to the “reliability of each semantic domain” [51], similarly showed high agreement (Table 1). Twenty-one discrepancies were resolved through discussions. Participants did not provide feedback on findings.

**Table 1:** Semantic domains, theme definitions, and intercoder agreement.

Semantic domains and theme definitions			Krippendorf cu-α
**Technology acceptance**			0.65
	Effort expectancy refers to the perceived ease or effortfulness with which one can navigate mobile apps and use wearable devices and seamlessly integrate them into one’s life.		
	Facilitating conditions are factors that can aid or impede the uptake or use of mobile apps and wearable devices. These include individual-related (eg, skills, predispositions, prior experiences) and technical-related (eg, infrastructure) factors.		
	Performance expectancy refers to the perceived usefulness or helpfulness of mobile apps and wearables in achieving desired health goals and behaviors.		
	Social influence refers to the perceived importance of significant others’ recommendations and approval of using mobile apps and wearables for health purposes.		
**Sentiment**			0.72
	Negative sentiment captures statements or remarks that indicate a sense of disapproval, criticism, or skepticism about any aspect of mobile technologies such as their worthiness, utility and impact, time and effort investment, and compatibility with one’s life.		
	Neutral sentiment captures statements or remarks that (1) are neither positive or negative in tone or (2) contain an equal number of positive and negative remarks.		
	Positive sentiment captures statements or remarks that indicate a sense of approval, praise, or certainty about any aspect of mobile technologies such as their worthiness, utility and impact, time and effort investment, and compatibility with one’s life.		
**Purpose of using mobile technology**			0.93
	Lifestyle and health management		
	Smoking cessation		

### Ethical Considerations

The study was deemed exempt by the National Institutes of Health Institutional Review Board on October 11, 2019, under Category 2: Research that only includes interactions involving educational tests, survey procedures, interview procedures, or observation of public behavior (§45 CFR 46.10(d)(2)); and Category 3: Research involving benign behavioral interventions (§45 CFR 46.10(d)(3)). The study was deemed exempt by ICF’s institutional review board on November 19, 2019, under Category 2. An amendment was approved by ICF’s institutional review board on February 26, 2020. All participants verbally consented to participate in the study prior to commencing audio recording. Only UserWorks had access to participants’ identifying information, whereas none of the authors did. UserWorks assigned enrolled participants identification numbers (eg, P1 and P2), which appeared on GoTo Meeting during the focus group sessions. Participants were not identified by name in the audio recordings or transcripts, only by their participant identification numbers. Participants received US $150 gift cards per focus group session.

## Results

### Overview

Sample characteristics appear in Table 2 and individual participant characteristics appear in Table S4 in Multimedia Appendix 1. Most participants were aware of lifestyle and health management uses of mobile technologies, with 22 participants (57.89%) reporting prior use of mobile apps or wearables for fitness, diet, and alcohol drinking, among other purposes (Table S4 in Multimedia Appendix 1). The number of participants using mobile apps was higher than those using wearables (55.26% vs 23.68%). Participants were able to name select apps (eg, Samsung Health App) and wearable devices (eg, Fitbit and iWatch). Conversely, only 7 (18.42%) participants had experience using mobile technologies for smoking cessation.

**Table 2:** Participant characteristics (N=38).

Characteristic		Values, n (%)
**Sex**		
	Female	20 (52.63)
	Male	18 (47.36)
**Race and ethnicity**		
	NH^a^ American Indian or Alaska Native	1 (2.63)
	NH Asian, Native Hawaiian, or Pacific Islander	3 (7.89)
	NH Black or African American	11 (28.94)
	Hispanic or Latino	6 (15.78)
	NH White	16 (42.10)
	NH Mixed	1 (2.63)
**Highest level of education**		
	Less than high school	3 (7.89)
	High school graduate	10 (26.31)
	High school equivalent	3 (7.89)
	Some college, no degree	18 (47.36)
	2-year associate degree	4 (10.52)
**Smoking frequency**		
	Every day	30 (78.94)
	Some days	8 (21.05)
**Quit timeframe**		
	7 days	11 (28.94)
	30 days	22 (57.89)
	6 months	5 (13.15)
**Smartphone operating system**		
	Android	21 (55.26)
	iOS	17 (44.73)

^a^NH: Non-Hispanic.

### Lifestyle and Health Management

Of 80 quotes relevant to lifestyle and health management uses of mobile technologies, 4 themes emerged with 55% of the quotes focused on facilitating conditions of use, 27.50% focused on the perceived usefulness of these technologies in helping participants attain their health goals, 13.75% focused on ease of using the technology, and 3.75% focused on the social influences themes (Table 3). All illustrative quotes appear in Table S5 in Multimedia Appendix 1.

**Table 3:** Distribution of number of quotes by technology acceptance and sentiment toward mobile technologies use for lifestyle and health management and smoking cessation purposes.

Themes^a^	Lifestyle and health management, n (%)				Smoking cessation, n (%)				Overall, n (%)
	Negative	Neutral	Positive	Total	Negative	Neutral	Positive	Total	
Effort expectancy	4 (36.36)	1 (9.09)	6 (54.54)	11 (13.75)	0 (0)	0 (0)	0 (0)	0 (0)	11 (8.39)
Facilitating conditions	14 (31.81)	28 (63.63)	2 (4.54)	44 (55.00)	3 (9.09)	29 (87.87)	1 (3.03)	33 (64.70)	77 (58.77)
Performance expectancy	6 (27.27)	1 (4.54)	15 (68.18)	22 (27.50)	10 (71.42)	1 (7.14)	3 (21.42)	14 (27.45)	36 (27.48)
Social influence	2 (66.66)	1 (33.33)	0 (0)	3 (3.75)	0 (0)	3 (75.00)	1 (25.00)	4 (7.84)	7 (5.34)
Total	26 (32.50)	31 (38.75)	23 (28.75)	80 (61.06)	13 (25.49)	33 (64.70)	5 (9.80)	51 (38.93)	131 (100.00)

^a^Column totals add to 100% within each theme, whereas overall row totals add to 100% across a semantic domain.

### Facilitating Conditions

The theme of facilitating conditions included factors that aided or impeded the use of mobile technologies. Participants cited the availability of health apps on their phones as a facilitator to their use.

Like most Android users, [I] have the Samsung Health [App] and I didn't even know it was on there until it just started telling me, hey, you're taking this many steps, so I actually started paying attention to it, and I think it has one for like monitoring how many hours you sleep, so that's about as far as I go.

P27

Lack of perceived health needs, limited awareness (especially of wearable devices), time and monetary costs, and technology-related factors (eg, battery drainage, software updates) impeded the use of mobile technologies for lifestyle and health management.

I just haven't come across [an app] or heard anybody talking about one that I thought would be relevant for my life, honestly.

P01

I ended up not keeping the app on my phone [for] very long. It just … drained my battery and it took up too much space and I had to keep Bluetooth on all the time. So, after about like two days, I just deleted it, but I still use the watch, you know, just to let it track my steps and everything.

P01

### Performance Expectancy

The performance expectancy theme overlapped with positive sentiment around the usefulness of mobile technologies for lifestyle and health management (68.18%). This was owed primarily to self-monitoring affordances and time-saving benefits.

I … was more active because of the fact that my steps were being tracked.

P20

Conversely, negative sentiment around the usefulness of mobile technologies for lifestyle and health management (27.27%) was attributed to factors such as skepticism about their accuracy and the availability of alternative methods to manage one’s health.

I often question the validity of some of those source options that you have … I think that [wearables like Fitbits and smartwatches] probably have more accuracy, but I always wonder if they're truly yielding the correct results. So, I’ve been hesitant to entertain those options.

P14

### Effort Expectancy

The effort expectancy theme overlapped with positive sentiment regarding the ease of using mobile health apps (54.54%) and was attributed to the constant access to smartphones and their reliability.

I've also used a couple [apps] for tracking behaviors, and I think they're a great idea … everyone's always on their phone, so you know, it's easier than writing things down, and you know, to set reminders and all that kind of thing. I think it just makes it easier for everyone.

P21

Effort-related negative sentiment usually arose in reference to wearables where participants cited device appearance, discomfort wearing them, and effort required to maintain a wearable device.

I’ve used a Fitbit before, just for like a little bit, but I never really got into it because I didn't like wearing something on my wrist.

P11

I'm just going to say, for me, if I were to get a Fitbit or something it's one more device that you have to maintain and use to take on and off.

P27

### Social Influence

Few quotes referenced the theme of social influences on mobile app and wearable use (3.75%) and centered primarily on friends and family recommendations for using specific health apps.

My sister got me on [the exercise app], and, I mean, I was using it for like, probably a couple of weeks, or a month or so [before I stopped].

P03

### Smoking Cessation

Fifty-one quotes focused on the use of mobile technologies for smoking cessation. Of those, 64.70% centered on facilitating conditions of use, 27.45% on their usefulness to help individuals quit smoking, and 7.84% on social influences themes (Table 3). None described their perceived ease of use. All illustrative quotes appear in Table S6 in Multimedia Appendix 1.

### Facilitating Conditions

Perceived health needs and prior use of mobile technologies for smoking cessation were primary facilitators of their use.

I've been smoking for, I'm gonna guess, like, over 10 years now. So, I'm actually looking to try to stop smoking because it's definitely not the route I want to go with my lungs and my health.

P09

I have, in all honesty, started using games to distract myself when I want to smoke.

P36

The main barrier was limited awareness of the availability of mobile technologies to aid in smoking cessation.

I never knew something existed out there. I just always thought … for … trying to quit smoking I thought the only resource was just going to some type of specialist for that.

P26

### Performance Expectancy

Despite lacking prior experiences with smoking cessation apps, the performance expectancy theme overlapped with negative sentiment regarding the usefulness of mobile technologies in aiding with quitting cigarette smoking (71.42%).

I've just come to realize that I'm just addicted to these cigarettes. So, if I'm … really addicted, then I don't know if the phone is gonna stop me or not.

P12

Skepticism stemmed from expectations that common features of health apps (eg, daily notifications) would not help them quit.

When I think of like, an app … the first thing that comes to mind, is just like, I'm gonna get like daily notifications. This is how you can quit or like, this is the reason that you should quit, or it’s like a bunch of negative stuff to me. So, that's the reason I haven't really tried actively looking for an app.

P08

### Social Influence

References to the social influence theme were rare (7.84%) and centered around peer recommendations and experiences with smoking cessation apps and devices. Social influences did not result in sustained use of these tools among participants.

One of my friends … does this app that helps … [you] quit smoking, and it counts down, like, how many cigarettes she’s allowed to have during the day … If I can remember the name, I’ll let you know, but that's the only other thing I've heard of.

P04

## Discussion

### Principal Findings

Young adults with low SES who smoke lack awareness of the existence of mobile technologies that can aid them in quitting cigarette smoking. Among the few who had knowledge of or experience with these tools, none reported sustained use or successful quit attempts aided by them. Regardless of prior knowledge or use, the majority were skeptical about the usefulness of mobile technologies as smoking cessation aids. Drawing upon their abundant experience with mobile health apps and wearables for lifestyle and health management, participants expressed negative sentiments about standard features such as daily notifications and generic health information. To realize the benefits of technology-based smoking cessation interventions, results suggest a need to raise awareness of how evidence-based mobile apps and sensors can support smoking cessation and employ user-centered designs to capitalize on accepted features in mobile health apps. This is especially important for populations disproportionally affected by smoking such as individuals with low SES who demonstrate high rates of interest in nonstandard smoking cessation services (eg, mobile apps and online chatting), particularly among younger individuals who smoke [52].

As digital natives, young adults are digitally literate and have higher smartphone ownership than any other age group [18,53]. However, few participants had any awareness of or experience with mobile technologies for smoking cessation, and among those who did have prior experience, they had nonsustained use, consistent with findings from previous work [54], and had difficulties recalling app names. Conversely, mobile apps and wearables for lifestyle and health management had name recognition and more participants were past or current users of such technologies. This is consistent with the well-established market size and use trends of lifestyle mobile apps and wearables compared with those for smoking cessation [55,56], with research showing low rates of awareness and use of smoking cessation apps among both health care providers and those who smoke [57,58]. This suggests that awareness and use of smoking cessation apps may be lower among the general population and that the idea of mobile apps and sensors as a smoking cessation approach is not yet as widespread as it is for lifestyle and health management [59]. To promote awareness and use of technology-based smoking cessation services, especially among socioeconomically disadvantaged individuals who smoke, health and medical professionals should facilitate referrals through electronic health records [25,26,60], partnerships with organizations outside traditional health care settings (eg, churches) [61], and insurance coverage for behavioral interventions following successful precedents [62]. Finally, tobacco cessation apps can be made readily available on smartphones as part of standard apps usually present on iPhones or Androids. App marketplaces (eg, Apple App Store) can recommend smoking cessation apps (and compatible wearables and sensors) within their health app categories.

Few participants had prior experiences with smoking cessation apps and quit attempts using them were unsuccessful. This may be a consequence of many smoking cessation apps relying on simple features (eg, calculators and calendars), not adhering to clinical practice guidelines, and not being empirically validated [31,63-65]. Among those with no prior experiences, we report skepticism about their usefulness in helping them quit smoking. Perceptions of performance are critical since negative beliefs may decrease uptake and sustained use of these apps [34,35]. Efforts should be directed toward designing evidence-based mobile-based smoking cessation interventions and using proven marketing strategies to promote such tools. For instance, testimonials and app ratings could demonstrate user satisfaction and app effectiveness for potential users [66].

High familiarity and use of mobile apps and wearables for lifestyle and health management can be a double-edged sword within a smoking cessation context. Participants’ experiences, which were largely positive with many participants commenting on their usefulness and effortlessness, could be a facilitating condition for using mobile technologies for smoking cessation [30]. Indeed, past behaviors are predictive of future behaviors [37]. Furthermore, smoking cessation mobile apps can integrate user-accepted features present in lifestyle and health mobile apps. For example, participants had favorable opinions of behavioral and physiological tracking and personalization consistent with prior research that identified such features as important for smoking cessation apps [57,67]. Additionally, one participant reported using mobile games to distract from smoking, consistent with current design features in cessation apps (eg, National Cancer Institute’s quitSTART app) and with prior research exploring the use of games for smoking cessation [68]. Conversely, negative experiences with lifestyle and health management digital tools can hinder the uptake and use of smoking cessation apps. Our results show that participants drew on their prior experiences with mobile health technologies when formulating opinions on smoking cessation apps. For example, some participants voiced concerns about the frequency and usefulness of notifications and the provision of generic health information. These results suggest, first, the need for user-centered designs to promote acceptance and use of smoking cessation mobile apps [69]. Second, smoking cessation interventions should fully use the capabilities of mobile technologies. Research on just-in-time adaptive interventions to deliver personalized support to users in real-time is an example of such efforts [70]. Finally, as part of efforts to promote smoking cessation apps, public health professionals should aim to distinguish smoking cessation apps from those for other lifestyle behaviors and health conditions that might be perceived as ineffective or effortful.

Facilitating conditions for using mobile technologies were qualitatively different for lifestyle and health management apps than for smoking cessation. This is owed to the different stages at which the former and the latter are spread in society [59]. Noteworthy, costs and other well-documented barriers to using mobile technologies (eg, privacy) were not frequently reported in our results, suggesting there may be a shift in perceptions as mobile technologies become more mainstream. However, other facilitating conditions, especially those that are structural in nature, apply equally to lifestyle and health management apps and to smoking cessation apps. For example, individuals with less disposable income might be less inclined to spend money on health apps, lack of cultural inclusivity can hinder uptake and sustained use of health apps, and lack of awareness could be attributed to limited health app prescriptions by health care providers [71,72]. While our participants emphasized barriers to smoking cessation mobile app use that have been documented in the literature among other populations, these barriers remain important when designing and recommending mobile health interventions for socioeconomically disadvantaged populations [71,73]. Future research is needed to further uncover individual and structural factors that may impede the use and efficacy of smoking cessation mobile apps. Additionally, future research should include individuals of both low and high SES to get a complete and nuanced picture of barriers to using smoking cessation mobile apps. It is also important to distinguish factors associated with the uptake and use of wearable devices as participants reported factors associated with their acceptance that differed from those for mobile apps. Wearables allow for the passive sensing of behaviors related to smoking, which can be used to tailor cessation support [2].

### Strengths and Limitations

This study has several strengths and limitations. Although prior studies assessed perceptions of mobile technologies for smoking cessation among populations disproportionally affected by smoking (eg, Blacks/African Americans and individuals with mental illness) [74,75], this is the first study, to our knowledge, to characterize perceptions among young adults with low SES who smoke. We recruited a diverse group of participants with roughly ≤50% of the participants from one race or sex. We conducted the focus groups digitally due to the COVID-19 pandemic, which facilitated nationwide recruitment but could have hampered discussions. We recruited participants with select inclusion criteria whose opinions and preferences may not be representative of others who smoke. Specifically, as digital natives, our participants are likely more accepting of and skilled in using digital technologies and more inclined to use these technologies for health purposes than others who are older [53]. Last, the number of participants was not equal across the 12 focus groups because of scheduling difficulties.

### Conclusions

The utility of mobile technologies in aiding smoking cessation rests upon user acceptance and adoption of such technologies. Our results show that young adults with low SES who smoke had limited awareness of the existence of digital health tools for smoking cessation and questioned their effectiveness. User-centered approaches are needed to ensure smoking cessation apps meet the needs of those who smoke and capitalize on the desired features of health and lifestyle apps. Furthermore, efforts are needed to promote smoking cessation mobile interventions and increase their effectiveness.

### Disclaimer

The views expressed in this manuscript are those of the authors and do not necessarily represent the views of the National Institute on Minority Health and Health Disparities, the National Cancer Institute, the National Institutes of Health, or the US Department of Health and Human Services. The authors attest that there was no use of generative artificial intelligence technology in the generation of text or other information content of this manuscript.

## Appendix

Multimedia Appendix 1 Supplementary tables.

## Abbreviations

COREQ: consolidated criteria for reporting qualitative research
SES: socioeconomic status
