# Supplementary material for: Perceptions of the Use of Mobile Technologies for Smoking Cessation: Focus Group Study With Individuals of Low Socioeconomic Status Who Smoke
Source: JMIR Form Res. 2024 Oct 11;8:e58221. doi: 10.2196/58221 (PMC11512139; doi:10.2196/58221)
Supplement: Multimedia Appendix 1 [file formative_v8i1e58221_app1.docx]

**Table S1.** Consolidated criteria for reporting qualitative studies (COREQ). [1]

| **Topic** | **Guide Questions/Description** | **Page No.** |
| --- | --- | --- |
|  |  |  |
| Domain 1: Research team and reflexivity |  |  |
|  |  |  |
| *Personal Characteristics* |  |  |
| Interviewer/facilitator | Which author/s conducted the interview or focus group? | 7 |
| Credentials | What were the researcher’s credentials? | 1 |
| Occupation | What was their occupation at the time of the study? | 7 |
| Gender | Was the researcher male or female? | 7 |
| Experience and training | What experience or training did the researcher have? | 8 |
|  |  |  |
| *Relationship with participants* |  |  |
| Relationships established | Was a relationship established prior to study commencement? | 8 |
| Participant knowledge of the interviewer | What did the participants know about the researcher? | 8 |
| Interviewer characteristics | What characteristics were reported about the interviewer/facilitator? | 8 |
|  |  |  |
| Domain 2: Study design |  |  |
|  |  |  |
| *Theoretical framework* |  |  |
| Methodological orientation and Theory | What methodological orientation was stated to underpin the study? | 8 |
|  |  |  |
| *Participant selection* |  |  |
| Sampling | How were participants selected? | 7 |
| Method of approach | How were participants approached? | 7 |
| Sample size | How many participants were in the study? | 7 |
| Non-participation | How many people refused to participate or dropped out? | 7 |
|  |  |  |
| *Setting* |  |  |
| Setting of data collection | Where was the data collected? | 7 |
| Presence of non-participants | Was anyone else present besides the participants and researchers? | 8 |
| Description of sample | What are the important characteristics of the sample? | 18 |
|  |  |  |
| *Data collection* |  |  |
| Interview guide | Were questions, prompts, guides provided by the authors? Was it pilot tested? | 7 |
| Repeat interviews | Were repeat interviews carried out? If yes, how many? | 7 |
| Audio/visual recording | Did the research use audio or visual recording to collect the data? | 8 |
| Field notes | Were field notes made during and/or after the interview or focus group? | 8 |
| Duration | What was the duration of the interviews or focus group? | 7 |
| Data saturation | Was data saturation discussed? | 7 |
| Transcripts returned | Were transcripts returned to participants for comment and/or correction? | 8 |
|  |  |  |
| Domain 3: Analysis and findings |  |  |
|  |  |  |
| *Data analysis* |  |  |
| Number of data coders | How many data coders coded the data? | 8 |
| Description of the coding tree | Did authors provide a description of the coding tree? | 17 |
| Derivation of themes | Were themes identified in advance or derived from the data? | 8 |
| Software | What software, if applicable, was used to manage the data? | 8 |
| Participant checking | Did participants provide feedback on the findings? | 8 |
|  |  |  |
| *Reporting* |  |  |
| Quotations presented | Were participant quotations presented to illustrate the themes/findings? Was each quotation identified? | 9-12 |
| Data and findings consistent | Was there consistency between the data presented and the findings? | 9-12 |
| Clarity of major themes | Were major themes clearly presented in the findings? | 9-12 |
| Clarity of minor themes | Is there a description of diverse cases or discussion of minor themes? | 9-12 |

**Table S2.** Moderation guide.

| **Questions** |
| --- |
| 1. I’d like to begin by having each of you tell us about how you started smoking. 2. Have you heard of mobile apps to help manage your health? What are your thoughts around these? Have you ever used one before? Why? Why not? 3. Think about the health app you use the most. What do you like about this app? Why do you use this app more than other apps on your phone? |
| 1. Have you ever used a mobile sensor, a device that syncs with your phone, to monitor or track your health? For example, a Fitbit, blood glucose meter. If yes, what did you use? Why? If not, why not? |
| 1. Have you heard of smartphone apps for quitting smoking? Can you name the apps you know of? Have you ever used one before? Why? Why not? |

**Note S1.**

Consistent with literature, early age of initiation was common across participants, with the majority starting smoking before adulthood. Smoking initiation was fueled by well-documented factors, mainly peer and family influences, perceived social norms, and curiosity. [2,3] Several participants emphasized the unplanned and experimental nature of early cigarette smoking, which reflects mechanisms of risky behaviors in adolescence. [4] Reasons for sustaining smoking included habitual smoking, addictive smoking, and negative or positive affect smoking (e.g., stress relief), which reflect underlying psychology of smoking behavior. [5] Perceived health risks were an underlying reason for participants’ willingness and desire to quit smoking. [6]

**Table S3.** Themes and illustrative quotes of smoking behaviors.^a,b^

| **Theme** | **Quotations** |
| --- | --- |
| Peer & family influence | P30: “It kind of started … around my friend group in high school … I’d probably kind of fall into the category of peer pressure. My friends are doing it, they probably got into it too.” |
|  | P29: “I was in high school, like early high school, and a lot of my friends were doing it as well as our parents.” |
|  | P13: “I started smoking because I was around a lot of my friends who smoked, and then my dad had cigarettes around all the time because he smoked as well. So, I kind of just picked it up those ways.” |
|  | P01: “My stepdad … he smoked, and he always kept like a carton in the freezer. So, it was easy for me to go out there and [grab] a pack.” |
|  | P28: “First year of high school, there were a few friends doing it … but I didn't really get into it, but my cousin constantly did it, and when I was spending time with her, she was like … you have friends around you doing it and you know I do it and … she let me try it … From there, I ended up doing it, like everybody else around me.” |
|  | P16: “I started smoking at a young age, a lot of peer pressure, just hanging around a lot of people that were smoking … just being around people that [smoked], so I kind of got peer pressured into it at a young age.” |
|  | P02: “I started with primarily … being around family.” |
|  | P08: “My mom, she smoked for a long time and so I watched her smoke, and I was interested and kind of stole a cigarette from her one day and then I started smoking from there.” |
|  | P27: “I probably picked up the habit, it was in college and … just having friends … who were smokers just kind of rubbed off on me.” |
|  | P25: “When I was around an older friend, and I would always watch him smoke, and then I just asked for one.” |
|  | P15: “I started probably when I was about 15 or 16. You know, with my friends.” |
|  | P38: “Honestly, it was when I was young. Honestly, it was peer pressure. So, that’s what happened.” |
|  | P36: “I started when I was 14 and I stole cigarettes for my grandpa, who I was living with.” |
|  | P31: “Well actually it was my big brother who smoked, and I always followed his … footsteps. So, then I started smoking.” |
|  | P11: “I started smoking when I was like 16 or 17 because my friend was.” |
|  | P32: “I started smoking around high school because of peer pressure.” |
|  | P35: “I started around high school as well, and I was just following … my best friends because they started it.” |
|  | P33: “It was a little late. I didn't really start until I was like 17 … it was more so just like my friends and my girlfriend at the time … smoked a lot.” |
|  | P02: “[I] started smoking probably around 16. I have a brother that has been [smoking] … since he was about like 13 or 14. It is just family influence, I guess.” |
|  | P03: “It was just … family influenced … I just grew up with it.” |
|  | P14: “A lot of it played in part to my two older sisters smoking and being exposed to that and seeing them do that.” |
|  | P16: “I also started young smoking. My mom smoked; my dad smoked … Seeing everyone else do it.” |
|  | P15: “I started … at a young age as well, you know, hanging out with friends, and things like that.” |
|  | P20: “I started smoking cause … friends around me were and my big brother did … He bought me cigarettes and gave me cigarettes.” |
|  | P04: “I think it was my junior or senior year, I guess it started picking up more because my friends started doing it more. So, it went from there.” |
|  | P06: “Most of my family members smoke and … I'm just used to it.” |
|  | P11: “I started when I was like 15 or 16 … because my like best friends were smoking.” |
|  | P13: “I started smoking, I think, like when I was younger, and like I had friends that smoked and then my father, smoked, and it was just there.” |
|  | P12: “Started when I was 19 and everybody that I was going to college with was [smoking].” |
|  | P23: “I started smoking in high school and it was just around like a group of friends, and I was just kinda influenced that way. And that's just like how it started, and I guess I just picked up from there.” |
|  | P22: “I started smoking in middle school. I did have some family members that smoked in my household which made it a little easier and more attractive.” |
|  | P09: “It was the beginning of my high school years … Well, every high school has the smoker group, and I ended up hanging out with the wrong group, the wrong crowd, and went out to the smoker's section, and inevitably started smoking cigarettes from there on out.” |
|  | P03: “I'm the oldest of nine kids and I can honestly say all my brothers and sisters smoke … even the younger ones are starting to … smoke now.” |
|  | P03: “My mom and my stepdad, they've been smoking for over 20 plus years … and my … grandma and grandpa passed away, which are both my mom’s parents.” |
| Perceived social norms | P29: “Growing up and seeing our parents doing it, we thought it was normal.” |
|  | P26: “It was just socially acceptable, and I would be more influenced to smoke a lot, like a couple of more cigarettes.” |
|  | P02: “It was acceptable, you know.” |
|  | P15: “[I started smoking] thinking it was cool and stuff like that.” |
|  | P34: “It just [seemed] socially acceptable.” |
|  | P15: “I just thought it would be cool.” |
|  | P21: “[I] started smoking when I was 13 years old. It was just kinda the thing that everybody did … I just kind of intended to go along with everyone else and here I am years later.” |
|  | P24: “My mom has smoked the majority of like, my entire life. So, at first, I was really against it. And then, I traveled to Uganda, and I also saw the culture of people smoking. Like, it wasn't just my mom who I was seeing.” |
|  | P28: “I was curious.” |
| Unplanned nature of smoking initiation | P16: “I just picked up a cigarette one day and tried it out.” |
|  | P06: “When I was like 14 or 15, I started to experiment … [with] the leftovers that my brothers and my parents left there, and I started smoking.” |
|  | P13: “[I] just wanted to try it.” |
|  | P07: “Just … picked up a half of a cigarette butt off a playground one day … Without hesitating, I don't know why I did it, just did it.” |
|  | P08: “My mom, she used to be a smoker, and I found her pack of cigarettes … me and my friend we decided to smoke it, and my friends started coughing and I didn't … ever since then, I smoked every day.” |
|  | P10: “I was just kinda curious.” |
| Habitual smoking | P29: “We just started doing it, and, yeah, it just kinda never stopped.” |
|  | P01: “I just kept, kept smoking.” |
|  | P30: “It became kind of the daily routine.” |
|  | P02: “I think it was just, like, engrained in my subconscious.” |
|  | P05: “I just originally started smoking socially, at gatherings and after class and whatnot, and it kinda just built to a habit from there.” |
|  | P07: “Sometimes I'll look over and all of a sudden, I have a cigarette in my hand, and I don’t even remember getting it. Sometimes it's just instincts, you just kind of see your pack there and you grab it.” |
|  | P16: “I have a habit as soon as I wake up in the morning and get my kids ready for school. No matter how late I am I have to smoke a cigarette in the morning, and I think it's really disgusting habit and I don't like it but it's something that I'm not strong enough to break.” |
| Addictive smoking | P25: “I've been addicted … since that [first] one. It was like a refreshing feeling … like winter fresh and I've been smoking since.” |
|  | P15: “I tried [smoking], and you know, I got addicted to it, and couldn't quit from there.” |
|  | P13: “I got kind of addicted over time.” |
|  | P07: “I just fell into like a hole. Lot of times, smoking, I've been doing it since, probably around 10 years old.” |
|  | P10: “I just picked up a cigarette last year and I'm hooked now.” |
|  | P09: “I've tried to quit in the past before, and I have [quit] cold turkey and … inevitably came back to it.” |
|  | P01: “During the day, when I'm at work there are certain times that … my body just decides that it's time for a cigarette, and if I don’t get one … I'm just miserable.” |
| Negative affect | P28: “It became one of those stress reliever things, like, anytime something stressful happened. I just, you know, need it.” |
|  | P16: “[I started smoking because of] just having a lot of stress at a young age.” |
|  | P16: “Just being young and having a lot of problems and a lot of stresses at a very young age … Being around people that use cigarettes for stress and always going to cigarettes for stress, it kinda just rolled over to me.” |
|  | P18: “When you're stressed out in life … you just pick up a cigarette and it does something to you … It calms you.” |
|  | P14: “Work or professionally related, feeling mainly stress and feeling like my life is just crazy and constantly like, go, go, go, go … Smoking for me … it's just almost like a release and reward thing where I can just go and sit and smoke and relax and not think for the time that I am smoking that cigarette.” |
| Positive affect | P27: “[I started smoking] and I actually liked it.” |
| Perceived risks | P03: “For the longest … I didn't smoke … because I've seen how it made my mom and my grandma and everybody in my family, like, they couldn't breathe, they couldn't do nothing for real.” |
|  | P18: “Smoking makes you old. It’s bad for your health and it’s expensive.” |
|  | P18: “[Smoking] is not healthy.” |
|  | P03: “I remember going into the hospital … after my grandma had had surgery before she passed away and they had sucked out all the tar in her in her body … My mom was like, that's all the tar from cigarettes that she's been smoking all these years … My mom has said so many times, she wants to stop smoking, because she is starting to have part complications … and she has to use a breathing machine … She can barely walk up the stairs without losing breath. Same with my stepdad.” |

^a^Total number of quotes on smoking initiation and use = 73.

^b^References to early smoking initiation were evident either implicitly or explicitly in illustrative quotes for various themes and thus are not presented as a separate theme.

**Table S4.** Participant characteristics.

| **ID** | **Sex** | **Race and ethnicity^a^** | **Highest level of education^b^** | **Smoking frequency** | **Quit timeframe** | **Smartphone operating system** | **Prior experience** | | |
| --- | --- | --- | --- | --- | --- | --- | --- | --- | --- |
|  |  |  |  |  |  |  | **Type of mobile technology** | **Health and lifestyle use** | **Smoking cessation use** |
| P01^*^ | Female | NH White | Some college, ND | Every day | 30 days | Android | Apps and smartwatch | Exercise/fitness |  |
| P02^*^ | Male | Hispanic/Latino | Some college, ND | Every day | 30 days | Android |  |  |  |
| P03 | Female | Hispanic/Latino | HS incomplete | Every day | 7 days | Android | Apps | Exercise/fitness |  |
| P04^*^ | Female | NH White | Some college, ND | Some days | 30 days | Android | Apps | Drinking water; exercise/fitness |  |
| P05 | Male | NH White | Some college, ND | Every day | 7 days | Android | Apps | Exercise/fitness; dieting |  |
| P06 | Male | NH Black/AA | Some college, ND | Every day | 30 days | Android |  |  |  |
| P07 | Male | NH Asian, NHPI | HS equivalent | Every day | 30 days | iOS |  |  |  |
| P08^*^ | Female | Hispanic/Latino | Some college, ND | Every day | 30 days | iOS | Apps and smartwatch | Exercise/fitness; sleep | Yes |
| P09 | Male | NH White | HS equivalent | Every day | 30 days | iOS | Smartwatch | Unspecified |  |
| P10^*^ | Male | NH White | HS graduate | Every day | 7 days | Android | Apps | Alcohol consumption | Yes |
| P11^*^ | Female | Hispanic/Latino | Some college, ND | Every day | 30 days | iOS | Apps and wearables | Alcohol consumption; drinking water; exercise/fitness; habits |  |
| P12^*^ | Male | NH White | Some college, ND | Every day | 7 days | Android |  |  |  |
| P13^*^ | Female | NH White | HS graduate | Every day | 30 days | Android |  |  |  |
| P14^*^ | Female | NH White | Two-year AD | Some days | 30 days | iOS | Apps | Exercise/fitness; meditation; telehealth |  |
| P15^*^ | Female | NH Black/AA | HS incomplete | Some days | 30 days | Android | Apps and smartwatch |  | Yes |
| P16^*^ | Female | AIAN | HS incomplete | Every day | 30 days | iOS | Apps | Dieting; exercise/fitness |  |
| P17^*^ | Male | NH Black/AA | HS graduate | Every day | 30 days | Android | Apps |  | Yes |
| P18 | Female | NH Black/AA | Some college, ND | Every day | 7 days | iOS | Apps | Dieting; exercise/fitness |  |
| P19 | Female | NH White | HS equivalent | Every day | 7 days | Android |  |  |  |
| P20 | Female | NH White | HS graduate | Every day | 7 days | Android | Apps and smartwatch | Exercise/fitness | Yes |
| P21 | Female | NH White | Some college, ND | Every day | 7 days | iOS | Apps and smartwatch | Dieting |  |
| P22 | Female | NH White | Some college, ND | Every day | 30 days | iOS | Apps |  | Yes |
| P23 | Male | NH White | Some college, ND | Every day | 7 days | iOS |  |  |  |
| P24 | Female | NH Black/AA | Some college, ND | Every day | 30 days | Android | Apps | Exercise/fitness |  |
| P25 | Female | NH Black/AA | HS graduate | Some days | 30 days | Android |  |  |  |
| P26 | Male | NH Black/AA | Some college, ND | Every day | 30 days | iOS |  |  |  |
| P27 | Male | Hispanic/Latino | Some college, ND | Some days | 30 days | Android | Apps | Exercise/fitness; sleep |  |
| P28 | Female | NH White | Two-year AD | Every day | 30 days | Android | Apps and smartwatch | Exercise/fitness; unspecified purposes |  |
| P29 | Female | NH White | Some college, ND | Every day | 30 days | iOS | Apps | Unspecified purposes |  |
| P30 | Male | NH Asian, NHPI | HS graduate | Some days | 6 months | Android | Apps and smartwatch | Exercise/fitness |  |
| P31 | Male | NH Black/AA | Some college, ND | Every day | 6 months | Android | Apps | Exercise/fitness | Yes |
| P32 | Female | NH Asian, NHPI | HS graduate | Every day | 30 days | iOS |  |  |  |
| P33 | Male | Hispanic/Latino | Two-year AD | Some days | 6 months | iOS | Apps and smartwatch | Dieting; exercise/fitness |  |
| P34 | Male | NH Black/AA | Two-year AD | Every day | 6 months | Android | Apps | Exercise/fitness; habits |  |
| P35 | Female | NH Black/AA | HS graduate | Every day | 30 days | iOS | Apps | Dieting; exercise/fitness |  |
| P36 | Male | NH White | Some college, ND | Every day | 7 days | Android |  |  |  |
| P37 | Male | NH Black/AA | HS graduate | Every day | 6 months | iOS |  |  |  |
| P38 | Male | NH Mixed | HS graduate | Some days | 7 days | iOS |  |  |  |

^a^NH = Non-Hispanic, AA= African American, NHPI = Native Hawaiian/Pacific Islander, AIAN= American Indian/Alaska Native. Participants who identified as Hispanics or Latinos were considered as such regardless of race.
^b^HS = high school, ND = no degree, AD = associate degree.

^*^Participated in two focus groups.

**Table S5.** Themes and illustrative quotes of smokers’ perceptions of using mobile technologies for health and lifestyle management.^a^

| **Themes** | **Subthemes** | **Quotations** | **Sentiment** |
| --- | --- | --- | --- |
| Facilitators and Barriers | Prior Experiences | P29: “I've definitely [used] like health and fitness apps, but I guess I just never really felt like taking it a step further.” | Negative |
|  |  | P16: “I've used a fasting app and I've used an exercising app … the Fast Habit app. That’s the one I used for the exercising.” | Neutral |
|  |  | P04: “I’ve used [a mobile app] for … walking distance and … heart rate and stuff like that.” | Neutral |
|  |  | P35: “I use a couple of [mobile health apps] just to track my health … count calories to watch what I'm eating. Yeah, I use a couple of them … just mainly for like working out or watching what I'm eating.” | Neutral |
|  |  | P11: “I've used apps before but also for like health, fitness stuff like My Fitness Pal … I've also used apps to quit drinking … apps for like making your new habits.” | Neutral |
|  |  | P33: “I used to use one a … year ago, when I was on keto to like track … what I ate. But that's like the only kind of health app I ever used.” | Neutral |
|  |  | P31: “As part of the mobile apps … I did use them for exercising and things like that.” | Neutral |
|  |  | P34: “I've used my phone to monitor my heart rate and … to like monitor [my] stress level.” | Neutral |
|  |  | P34: “I’ve used apps for like working out and … to create new habits.” | Neutral |
|  |  | P03: “I’ve used one of those walking apps. You know that like tracks how many steps you take in a day … I think it's called like Sweat Coins.” | Neutral |
|  |  | P01: “I have like the knock off [wearable], it wasn’t a Fitbit, it was just a cheap one.” | Neutral |
|  |  | P16: “I actually have a couple on my phone, but they're more like exercise apps and diet tracking apps … I have a couple of my phone, but it's just more like to eat healthy and lose weight and stuff.” | Neutral |
|  |  | P16: “[The mobile health app I used] was to … track my steps or, like, lose weight, and I would just … go into the app, start it, and put it in my pocket, and just walk … It was supposed to track my steps and how much I was walking.” | Neutral |
|  |  | P20: “Back when I had … an iPhone I had an iWatch and I tracked my steps and my heart rate and you know, things of that nature.” | Neutral |
|  |  | P21: “I've used some before. I've used mostly weight loss apps.” | Neutral |
|  |  | P11: “I don't remember the specific … names of them, but I know there was one for like dry January, so [it] kind of just tracked … your alcohol consumption and would … show you how much money you were saving … There's like workout apps or mindfulness, meditation, yoga, stuff like that.” | Neutral |
|  |  | P11: “I've definitely tried before … There was an app, I don't remember, I think it was like Plant Nanny … you like would drink cups of water and it would … help you keep track of how much water you're drinking throughout the day.” | Neutral |
|  |  | P09: “For me, only my iWatch, I guess.” | Neutral |
|  |  | P18: “[I’ve used] like a stepping app on my phone. Yeah, like how many steps a day I take, you know? And yeah, that’s the only one.” | Neutral |
|  |  | P11: “I like the idea of mobile apps for that. I have tried a few … I think there are some that are really good and some that aren't.” | Positive |
|  |  | P12: “[Wearables are] not something I ever wanted to spend money on.” | Negative |
|  | Time and Monetary Costs | P22: “No reason for me in particular … I'm not the kind of person that would probably go out and like purchase [a wearable], but if I got it for Christmas, I would probably use it.” | Negative |
|  |  | P23: “I think that's like something where that if I got [a wearable] as a gift I would use it, but I wouldn't go out and get that for myself.” | Negative |
|  |  | P17: “I just didn’t have the time to [use a wearable].” | Negative |
|  |  | P14: “Time is a factor [for not using wearables].” | Negative |
|  |  | P11: “I think the free ones are obviously better because … paying for stuff is sometimes kind of hard.” | Neutral |
|  |  | P29: “I don't know [about] like purchasing something to … track my health, but it's something I'm open to. I've just never really done it.” | Neutral |
|  | Awareness and Knowledge | P13: “I heard about them [in a research setting] that even now, I really haven't really looked into [mobile health apps] at all or been kind of motivated to even look at them, I guess.” | Negative |
|  |  | P05: “I've heard of a few [mobile health apps] but I haven't I haven't really used them other than just like the step counters … and whatnot.” | Neutral |
|  |  | P24: “I had only heard of like exercising apps, like things to help you lose weight.” | Neutral |
|  |  | P10: “I didn't know [wearables were] available. I've heard of the apps … they give updates on your health. Never ones that are like connected to your body at all, or like sensors, or anything.” | Neutral |
|  |  | P19: “I didn't even know … [sensors were] a thing.” | Neutral |
|  |  | P12: “I've never really heard of any apps like that either … if it works, it works. So, it could be a cool idea.” | Positive |
|  | Technical Factors | P01: “I ended up not keeping the app on my phone [for] very long. It just … drained my battery and it took up too much space and I had to keep Bluetooth on all the time. So, after about like two days, I just deleted it, but I still use the watch, you know, just to let it track my steps and everything.” | Negative |
|  |  | P03: “[The exercise app] just got to be too much, and, you know, was just taking up too much storage in my phone, so [I] just deactivated it.” | Negative |
|  |  | P17: “I don’t ever have the space on my phone to [use wearables or apps for wearables].” | Negative |
|  |  | P11: “I thought it was cool. I didn't like having to, like, charge it and I had a necklace [wearable] … Maybe like a longer battery [would work better with me].” | Neutral |
|  |  | P03: “I know that I've looked at … other apps and stuff before … they'd be easy to use, but then … after a week or two … you got to keep updating them.” | Neutral |
|  | Health Needs | P13: “I just really, personally, never really thought about it or thought that I needed [a wearable] … I’ve looked for stuff online for certain things, but never, like, for an app or anything like that.” | Negative |
|  |  | P28: “[Fitbit] is the only [wearable] I've ever considered, and since I have that, I don't feel the need to get something else.” | Negative |
|  |  | P03: “[The exercise app] just got to be too much for me … I'm not really worried about how much I walk anyways, because I know I walk a lot.” | Negative |
|  |  | P01: “I just haven't come across [an app] or heard anybody talking about one that I thought would be relevant for my life, honestly.” | Negative |
|  | Availability | P27: “Like most Android users, [I] have the Samsung Health [App] and I didn't even know it was on there until it just started telling me, hey, you're taking this many steps, so I actually started paying attention to it, and I think it has one for like monitoring how many hours you sleep, so that's about as far as I go.” | Neutral |
|  |  | P04: “My phone actually came with one. It's just like the Samsung Health app that I use just to count like steps and monitor how much like water I'm drinking, and little things like that, exercise.” | Neutral |
| Performance Expectancy | | P16: “As far as like, tracking the steps and stuff … I wasn't sure how accurate something like that would be on a phone as far as being on your wrist, like, the watches. So, those type of things I don't really bother with.” | Negative |
|  |  | P14: “I often question the validity of some of those source options that you have … I think that [wearables like Fitbits and smartwatches] probably have more accuracy, but I always wonder if they're truly yielding the correct results. So, I’ve been hesitant to entertain those options.” | Negative |
|  |  | P03: “My clothes, my clothes show me better than the apps do, to be honest with you. If I can fit it, then hey! If not, then, maybe I need to walk a little bit more, so, yeah.” | Negative |
|  |  | P03: “[The app I used was] called Sweat Coins … you can kind of like cheat the system … and like shake your phone … and it’ll like show that you walked so many steps … I could be sitting on a couch and just shake my phone and see, you know, ‘oh, you walked like 5, 5 steps’ or something and it like it rewards you … for however many steps you took on.” | Negative |
|  |  | P07: “I’ve seen [mobile health apps] around, but I’ve never tried to use one because I've never seen them being beneficial because they're always in your pocket, and I never always want to be looking at my phone. So, I guess, I mean, [I’m open to] giving it a try, giving it a go.” | Negative |
|  |  | P08: “I used to have like a Fitbit and stuff. It would tell you, like, your step count, like how much you're sleeping and things like that. I didn't find it super beneficial specifically for the reason that, like, I wasn't checking up on it, like I didn’t open the app every day. It's like, oh, this is how my steps look, or even when I get the notifications, like I just swipe up, just like kind of ignore them. So, it wasn't super beneficial to me.” | Negative |
|  |  | P24: “I’m more like I’d [want] to do a lot of research to know how [a wearable] really works. I don’t think I would just randomly go out and get it.” | Neutral |
|  |  | P30: “I have a Fitbit if that counts, and I found it to be really helpful. I use it to, kind of track my heart rate, exercise, this, and that … I've had a pretty good experience with those apps.” | Positive |
|  |  | P10: “I've used one [mobile health app]. Yes, and it seems to be going pretty well. I just got it two days ago … It's [called] … Easy Quit Drinking.” | Positive |
|  |  | P16: “The exercising app was really cool, I got to login like what I ate that day and exercise and my goal and stuff. So, it was really helpful.” | Positive |
|  |  | P33: “I use the Fitbit app, with like the Fitbit watch, that's like the health app that I use, and I like it a lot … because it like monitors like your health … It tells you like your what your pulse is, and like everything's like up to date, tells you how many steps you've taken in a day and things like that.” | Positive |
|  |  | P14: “I currently utilize a virtual … primary care physician doctor app that allows me to you actually go to the doctor via mobile app versus having to take off work and go in.” | Positive |
|  |  | P14: “I think that the Fitbits and the smartwatches, they're great. I just have never owned one or been in the market to spend the money on something like that.” | Positive |
|  |  | P14: “I utilize apps for working out and tracking my progress on a weekly and monthly basis, as far as what kind of workouts I'm doing, and the amount of time.” | Positive |
|  |  | P14: “I also utilize certain health related applications on my phone on a day-to-day basis, but they're more so geared towards working out … I also have a lot of like meditative relating stress relief applications that I do find useful [and] that have [come] in handy … at work [when] I have back-to-back meetings.” | Positive |
|  |  | P14: “I do utilize Doctors on Demand, which is a mobile phone app that is covered through my health insurance through my employer … It’s just a great tool for me, to being able to actually have a mobile guideline, doctor appointment, versus having to take off of work to go in for something like a cold or something I think I just need antibiotics for.” | Positive |
|  |  | P20: “I … was more active because of the fact that my steps were being tracked.” | Positive |
|  |  | P21: “I had a Fitbit, and I was definitely more conscious of my steps, and you know the things I was doing because you know, it was being recorded and it gave me, you know, something to see my progress.” | Positive |
|  |  | P05: “I have been looking into some for helping out with dietary habits and things like that.” | Positive |
|  |  | P11: “I think that [mobile health apps] can be really helpful.” | Positive |
|  |  | P10: “I've used one [mobile health app]. There was a quit drinking app that I use for a while, and it worked pretty well.” | Positive |
|  |  | P18: “I’m always looking for stuff … because I'm a mom and I do have a kid and I … [use] workout apps where I can do workouts at home and stuff … just like apps like that, and then kind of like healthy apps with food and stuff.” | Positive |
| Effort Expectancy | | P27: “I'm just going to say, for me, if I were to get a Fitbit or something it's one more device that you have to maintain and use to take on and off.” | Negative |
|  |  | P11: “I’ve used a Fitbit before, just for like a little bit, but I never really got into it because I didn't like wearing something on my wrist.” | Negative |
|  |  | P24: “I tried a couple apps, and it was just hard … there wasn't really a reminder. It was just more like you dedicate the time to go into the app and decided to exercise. It wasn't like a reminder, like, oh, in two hours, we have our workout session.” | Negative |
|  |  | P02: “I feel like sometimes it's a little too complicated … it's not simple like just getting the app or just buying the device, you have to buy the device and the app, and people may not totally be committed to that idea, just yet.” | Negative |
|  |  | P11: “[The wearable I used] was more clunky than they are nowadays, but something that, like, wouldn’t be that noticeable would definitely be something that, like, would work better for me.” | Neutral |
|  |  | P28: “I've had health apps in the past as well I currently use the Fitbit one. But there have been other ones before that just helped trace the steps. But the Fitbit’s a bit easier ... and I think those are really convenient. I try to keep them a part of my health routine.” | Positive |
|  |  | P21: “I've also used a couple [apps] for tracking behaviors, and I think they're a great idea … everyone's always on their phone, so you know, it's easier than writing things down, and you know, to set reminders and all that kind of thing. I think it just makes it easier for everyone.” | Positive |
|  |  | P20: “I've heard of [mobile health apps], and I think that they're a great idea, because everyone uses their phone anyway, and everyone loves apps, that helps them deal with life and tasks [and makes them] more easy so I think it's a good way to try to get them going.” | Positive |
|  |  | P20: “I thought it was a great idea because and I like the idea of them, because … everyone uses a phone, you know, every day for their daily life, just for social media … I think it's a great way to include that in anything, really. If you want something done easier, have an app for it, you know?” | Positive |
|  |  | P11: “I have my phone on me all the time so it's like a lot easier than remembering to, like, carry on a journal, or something like that.” | Positive |
|  |  | P13: “I have my iPhone everywhere, so it'd be like really helpful to like, just remind me or like, I don't know, things like that would be nice.” | Positive |
| Social Influence | | P03: “My sister got me on [the exercise app], and, I mean, I was using it for like, probably a couple of weeks, or a month or so [before I stopped].” | Negative |
|  |  | P02: “[I haven’t gotten a wearable because] I think it’s kind of intimidating, to, just jump in, if I am not walking as much as like my girlfriend or her network is walking.” | Negative |
|  |  | P01: “I had a cousin who was on the keto diet, and she was telling me about using one to count, you know, the calories and points and stuff.” | Neutral |

^a^Participant ID appears before each quote for attribution.

**Table S6.** Themes and illustrative quotes of smokers’ perceptions of using mobile technologies for smoking cessation.^a^

| **Themes** | **Subthemes** | **Quotations** | **Sentiment** |
| --- | --- | --- | --- |
| Facilitators and Barriers | Awareness and Knowledge | P34: “I've heard of apps that usually link to like a number, or like, usually go to like a website, but never, nothing more than that … Nothing more than just more information.” | Negative |
|  |  | P36: “I’ve seen the apps to like stop smoking, but I’ve never used them.” | Neutral |
|  |  | P31: “I've heard of [smoking cessation apps], but I can’t tell you the names of all of them. There are a lot of them, I know.” | Neutral |
|  |  | P11: “I've never heard of an app that will specifically help stop smoking. I've heard of ones that will help stop just things in general, but not smoking specific.” | Neutral |
|  |  | P26: “I never knew … [smoking cessation apps] existed up until just before this session.” | Neutral |
|  |  | P26: “I never knew something existed out there. I just always thought … for … trying to quit smoking I thought the only resource was just going to some type of specialist for that.” | Neutral |
|  |  | P16: “I never knew there were apps … I never knew there were apps to quit smoking.” | Neutral |
|  |  | P02: “I didn’t know [that smoking cessation apps existed]. I didn’t really know.” | Neutral |
|  |  | P14: “I'd never came across any apps or … websites that help you quit smoking.” | Neutral |
|  |  | P37: “Yeah, I've never heard of an app like that [for smoking cessation].” | Neutral |
|  |  | P03: “I didn't think any [apps for smoking cessation] existed, to be truthfully honest … You see them commercials on TV for that one anti-cigarette campaign … that's about it, as far as I know.” | Neutral |
|  |  | P20: “I've seen apps … that track how many days and, you know, weeks, months, you know, et cetera. How long, but not … how to help you stop smoking.” | Neutral |
|  |  | P05: “No, this is actually the first time [I am hearing about smoking cessation apps].” | Neutral |
|  |  | P04: “This is … my first time hearing about [smoking cessation apps].” | Neutral |
|  |  | P13: “Yeah, I haven't heard of any [smoking cessation apps], to be honest with you. At all.” | Neutral |
|  |  | P11: “I think I've heard of apps that are similar, just kind of like to stop habits. I don't think it was like smoking specific. But it was to stop, you know, bad habits in general.” | Neutral |
|  |  | P24: “I personally, never thought there [were] any apps to quit smoking.” | Neutral |
|  |  | P10: “I've heard of a smoking app … it can play like a mini game during the craving to pass the time [and] get your mind off it.” | Neutral |
|  |  | P23: “I've never like even heard of like apps to quit smoking before … I’ve never, never heard of any of them before. So, it's kind of cool to be open to something like that.” | Positive |
|  | Prior Experiences | P10: “I downloaded one, it was called Easy Quit Smoking, and I didn't, I just didn't use it.” | Neutral |
|  |  | P15: “I’ve used on the past, I had to wear like this watch, and they track like every time I smoke and … how many times a day I smoked, and things like that.” | Neutral |
|  |  | P36: “I have, in all honesty, started using games to distract myself when I want to smoke.” | Neutral |
|  |  | P35: “[I have used health apps] but not so far with like, smoking or anything like that.” | Neutral |
|  |  | P11: “[I have used apps for health purposes] but not really smoking.” | Neutral |
|  |  | P34: “[I have used health apps but] not necessarily to quit smoking.” | Neutral |
|  |  | P15: “I had to like wear a watch, and then it tracks, like how many times I smoke a day, and then with the app, I just have to keep track of if I smoked like in the morning during breakfast, at home, or walking, things like that.” | Neutral |
|  |  | P20: “[The smoking cessation app I used] was, it was one of those basic, you know, calendar, apps, you know, that's track how long things pass.” | Neutral |
|  |  | P22: “There's this app that shows you every day how much healthier your body is, so like readings in the smoking. Like certain parts of your body would light up green, like, oh, this is way healthier, and this used to be more of a red color. I can’t remember the name of it, I think it was like, Quit Now, or Quit Smoking.” | Neutral |
|  |  | P31: “I did do an app for cigarette quitting some years ago where they text me all the time.” | Neutral |
|  | Health Needs | P15: “I think I can pretty much quit cold turkey … because I have before … but I wasn't really ready to quit. So, for me, I don’t feel like I need an app to help me quit, honestly.” | Negative |
|  |  | P09: “I've been smoking for, I'm gonna guess, like, over 10 years now. So, I'm actually looking to try to stop smoking because it's definitely not the route I want to go with my lungs and my health.” | Neutral |
|  |  | P18: “I smoke like, I think, I feel like too much now, and like I'm really trying to cut back. Because like I don’t want to smoke, but it’s just like a really bad habit that I smoke a lot of cigarettes.” | Neutral |
|  | Costs | P07: “[A friend used one and] said it helped him a lot, but it cost them a lot of money.” | Negative |
| Performance Expectancy | | P08: “I have an app, it's Smoke Free, well, I don't have it anymore, but it's to help you stop smoking. I deleted it because it wasn't super helpful to me.” | Negative |
|  |  | P31: “I did use one once to try to stop smoking … it didn’t work.” | Negative |
|  |  | P02: “I think I'm just a little concerned because I don't know how influential [an app] will be like to really get me to think about it, you know, and really help me stay accountable.” | Negative |
|  |  | P02: “I don't know how well something like that will work … I just have to see it a little more. See what's involved.” | Negative |
|  |  | P12: “I've just come to realize that I'm just addicted to these cigarettes. So, if I'm … really addicted, then I don't know if the phone is gonna stop me or not.” | Negative |
|  |  | P08: “When I think of like, an app … the first thing that comes to mind, is just like, I'm gonna get like daily notifications. This is how you can quit or like, this is the reason that you should quit, or it’s like a bunch of negative stuff to me. So, that's the reason I haven't really tried actively looking for an app.” | Negative |
|  |  | P17: “I heard about the apps. I tried that for a minute. But I dunno, I just couldn’t stop smoking.” | Negative |
|  |  | P18: “They have all kinds of stuff they show on TV, especially stuff about smoking and I just came across one … I feel like how is the app gonna help you stop smoking? Is it gonna like remind you? … What is it gonna do? Like it can’t do too much but remind you to not smoke, or I guess read up on stuff that makes you feel like you don't want to smoke anymore? I don't know how that would help someone stop smoking.” | Negative |
|  |  | P22: “I downloaded an app that was supposed to help with [quitting] smoking. It might've been even like Quit Smoking and [I] just remember it's showing you like, oh, it's been 24 hours since you smoked and look how much healthier your body is … you have this much more lung capacity.” | Negative |
|  |  | P10: “Yeah, so it's just one and I downloaded it, but didn't really use it … it was called Stop Smoking. It's the first app that came off of my Android.” | Negative |
|  |  | P03: “I mean, depending on how useful it is for me … If it's sitting here giving me alerts or something like, hey, you went four days without smoking today, let's make it number five. You know, and then it gives me like options and stuff to do … Or like, ideas of things to do, besides smoking, a cigarette.” | Neutral |
|  |  | P09: “It may be helpful.” | Positive |
|  |  | P10: “[I downloaded one] on my phone. Unused right now, but gonna try it soon.” | Positive |
|  |  | P10: “[If] there was something like [a smoking cessation app] … that actually would work very well.” | Positive |
| Social Influence | | P04: “One of my friends … does this app that helps … [you] quit smoking, and it counts down, like, how many cigarettes she’s allowed to have during the day … If I can remember the name, I’ll let you know, but that's the only other thing I've heard of.” | Neutral |
|  |  | P35: “I’ve heard of Quit Now, because actually one of my friends used it but that's pretty much the one [smoking cessation app] I know I'm aware of, of the other ones that are out there. I don't remember their names, but the main one is Quit Now.” | Neutral |
|  |  | P22: “A while ago, I just downloaded … [an app for smoking cessation] to check it out because a friend of mine had mentioned it to me.” | Neutral |
|  |  | P07: “One of my friends had ordered … an app … but you put all your cigarettes in … like a regular pack, and then on your phone, you set times throughout the day that that pack thing can unlock so you can have a cigarette … I thought that was a pretty cool concept because … you're kind of limited to how many cigarettes you smoke a day … That's kinda only thing I've ever seen or heard of.” | Positive |

^a^Participant ID appears before each quote for attribution.

**References**

1. Tong, A., Sainsbury, P., Craig, J. Consolidated criteria for reporting qualitative research (COREQ): a 32-item checklist for interviews and focus groups. Int J Qual Health Care 2007;19(6):349-57. PMID: 17872937.
2. Lakshmi, R., Romate, J., Rajkumar, E., George, A.J., Wajid, M. Factors influencing tobacco use behaviour initiation - From the perspective of the Capability, Opportunity, Motivation-Behaviour (COM-B) Model. Heliyon 2023;9(6):e16385. PMID: 37292260
3. Park, S.H. Smoking and adolescent health. Korean J Pediatr 2011;54(10):401-4. PMID: 22232621
4. Barrington-Trimis, J.L., Braymiller, J.L., Unger, J.B., McConnell, R., Stokes, A., Leventhal, A.M., Sargent, J.D., Samet, J.M., Goodwin, R.D. Trends in the Age of Cigarette Smoking Initiation Among Young Adults in the US From 2002 to 2018. JAMA Netw Open 2020;3(10):e2019022. PMID: 33021650
5. Tomkins, S.S. Psychological model for smoking behavior. Am J Public Health Nations Health 1966;56(12):Suppl 56:17-20. PMID: 5951586.
6. Yingst, J.M., Krebs, N.M., Bordner, C.R., Hobkirk, A.L., Allen, S.I., Foulds, J. Tobacco Use Changes and Perceived Health Risks among Current Tobacco Users during the COVID-19 Pandemic. Int J Environ Res Public Health 2021;18(4). PMID: 33673207
